# The Faces in Infant-Perspective Scenes Change over the First Year of Life

**DOI:** 10.1371/journal.pone.0123780

**Published:** 2015-05-27

**Authors:** Swapnaa Jayaraman, Caitlin M. Fausey, Linda B. Smith

**Affiliations:** 1 Department of Psychological and Brain Sciences, Indiana University, Bloomington, Indiana, United States of America; 2 Department of Psychology, University of Oregon, Eugene, Oregon, United States of America; Durham University, UNITED KINGDOM

## Abstract

Mature face perception has its origins in the face experiences of infants. However, little is known about the basic statistics of faces in early visual environments. We used head cameras to capture and analyze over 72,000 infant-perspective scenes from 22 infants aged 1-11 months as they engaged in daily activities. The frequency of faces in these scenes declined markedly with age: for the youngest infants, faces were present 15 minutes in every waking hour but only 5 minutes for the oldest infants. In general, the available faces were well characterized by three properties: (1) they belonged to relatively few individuals; (2) they were close and visually large; and (3) they presented views showing both eyes. These three properties most strongly characterized the face corpora of our youngest infants and constitute environmental constraints on the early development of the visual system.

## Introduction

Human face perception is remarkable for its precision and for its relevance to species-important tasks [1]. People can rapidly identify the faces of a large number of individuals, can categorize (known and unknown) faces by age, gender, and race, and can read the intentions and goals of others from their facial gestures [1–5]. A key theoretical dispute centers on the developmental origins of these advanced abilities: Are the specialized visual processes for human face perception evident in adults principally determined by genetic processes or are they the product of massive visual experience [6, 7]?

Two empirical facts and one reasonable assumption provide the key discussion points in this debate. The first fact is that very young infants, including newborns, show special sensitivities to and interest in human faces, a fact consistent with a visual system specialized to learn about faces from the start (see [8, 9]; and for review [10]). The second fact is that human face perception shows a long developmental course and is not fully mature until adolescence, a fact consistent with a possible role for visual experiences in the development of specialized face processes [11, 12]. The reasonable assumption is that faces are prevalent in the human visual environment through out life (e.g., [1, 7, 13–15]). However, understanding the role of experience in the development of face perception requires evidence on the quantity and quality of the *early* face experiences that begin the developmental process. Understanding how both innate propensities and visual experiences may constrain the long course of development also requires knowing whether the properties of face experience change systematically with development. Recent evidence using head cameras to study face exposure in 1 to 3 month olds indicates that faces of caregivers are certainly present in early visual environments [16] but does not address the question of whether those early experiences have unique properties nor whether the quality and quantity of face experiences change with age.

Normative estimates of awake and sleep times [17] indicate that by the time infants are 3 months old, they have been awake for about 800 hours; how much of that is faces? How we think about the role of visual experience in the development of specialized face processing depends on whether the answer is closer to 30 hours or 300 hours [18] as more limited experiences favor a stronger role for innate propensities. What are the distributional properties of the faces of different people in the infants’ environments? Early experiences that are dominated by the faces of a few individuals could play a role in narrowing the range of faces robustly discriminated and recognized [15, 16]. Expanding encounters with a larger number of people could drive later occurring changes in face perception as some have hypothesized [6, 19–23]. The visual properties of encountered faces are also relevant to understanding the role of experience in face processing. Do young infants receive primarily frontal views of faces that fit the hypothesized innate face template or more varied views [6]? Further, newborns have poor visual acuity [24–27] and so the relevant faces for very young infants may only be those faces that are close to the infant and thus visually large, presenting defining face information at low spatial frequencies. Over the first 6 months, infant acuity increases five fold [26, 27]; infant’s motor, social, and cognitive skills also change. Do the visual properties of faces in the environment change correspondingly? By normative estimates of sleep times as a function of age [17], as infants approach the end of their first year, they have been awake about 3100 cumulative hours. What are the cumulated opportunities to see faces during that time? Are the frequencies, properties, and proximities of faces in visually available scenes constant over the first year or do they change, and if so, is that change gradual or dramatic? These are open but important questions; understanding the role of experience in the development of face processing requires understanding the regularities in infant face experiences and how those regularities change with development.

The present study provides evidence on the quantity and quality of faces available to infants over the first year of life. Infant-perspective scenes were captured using a head camera. There has been growing use of head cameras in developmental research on visual environments and increasing understanding of the strengths and limitations of this method (for review see [28]). In brief, the head-camera captures the scene in front of the wearer’s head and thus the available environment as a function of the wearer’s stature, posture, and activity; the head camera does not capture the dynamics of looking behavior. Because eyes and heads are mostly but not always aligned (see [28]), the head camera is best-used for the collection and content analysis of large corpora of wearer-perspective scenes. This is the present use.

## Method

### Ethics statement

All experimental protocols and consent materials were approved by the Indiana University Institutional Review Board. Parents of all participating infants provided written informed consent prior to the experiment. The legal guardians of the individuals whose data or pictures appear in this manuscript have given written informed consent (as outlined in PLOS consent form) to publish these case details.

### Participants

The participants were 22 infants (11 female, 11 male) aged 1 to 11 months from middle class families in Monroe County, Indiana who were recruited through county birth records and community events. None of the infants in the present sample regularly attended center-based group daycare; in the United States only about 9% of infants under one year are in center-based group day care (although 50% are in non-parental care in their own home, with a relative, or in the home of another family) (see [29–31]).

### Head camera

**Fig 1:**
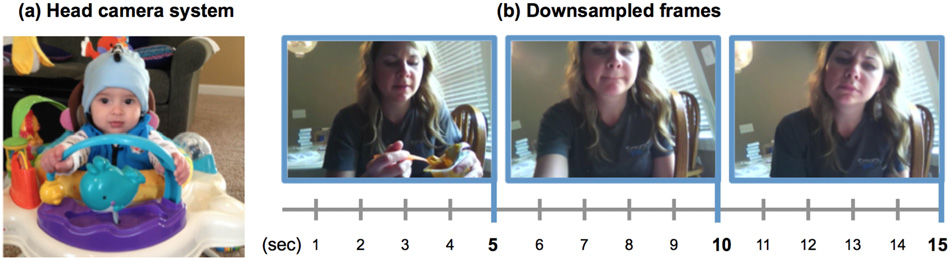
Head camera and image sampling. An infant wearing the head camera system (a). Frames at the rate of 1 every 5 seconds (the sampled rate for analysis) from the head camera (b).

Recording the availability of faces in infants’ everyday environments requires a method that is not disruptive of those daily environments. Accordingly, we used a wearable camera that was lightweight, cable-free, attached to daily-wear hats, and easy for parents to use. The head camera was the commercially available Looxcie 2. The camera has three critical properties relevant to this study: a very lightweight 22g body, built-in recording capacity, and a rechargeable non-heating battery. The camera measures 0.91” x 0.67” x 3.33”, has an f2.8 lens with a 75° diagonal field of view (41° vertical FOV, 69° horizontal FOV), and a 2” to infinity depth of focus. The FOV is a potential limiting factor as infant visual fields sensitive to point lights in the dark typically measure 90° horizontally and vertically [32–34]. Issues of limitations in camera FOV for detecting change in the environment have been discussed extensively by researchers using these methods [28, 35, 36]. The upshot is that the camera used in this study captures a broad view of what is directly in front of the child’s head—and thus content that is visually available to the child—but may miss peripheral information. Further, we also know from the growing number of infant head camera studies that the contents of these head scenes accurately predict infant performance in other domains—perceiving causal structure in actions [37], making decisions when navigating [38], learning object names [39, 40], and recognizing objects [41]. Each camera has a recording capacity of 3–4 hours of video at the rate of 30 frames per second. The camera was attached to a snug fitting hat as shown in Fig 1a. Parents were given two hat-camera systems.

### Procedure

In a pre-visit, parents were informed about the goal of the study, consent was obtained, and they were instructed on how to use the camera. A hat was selected and fit to the child. Subsequently, the materials were delivered to the infant’s home and the parents were reinstructed in the use of the camera. Parents were not told that we were interested in faces or social events but were told that we were interested in visual development and the typical range of visual experiences of their infant. They were asked to record during the infants’ waking hours and to try to capture four to six hours of video during a variety of daily activities when the infant was awake and alert. Because of the complexity and demands of parenting young infants, parents were given up to two weeks to complete their recording. Upon completion parents were debriefed and, consistent with the consent procedure, asked if they wanted any segments deleted.

### Final corpus and coding

The family of the youngest infant was only able to contribute 15 minutes of video. Total recording hours for the remaining infants ranged from 2.5 to 7 hours. With the youngest infant excluded, the hours of recording obtained from each infant did not vary systematically with age (R^2^ = .12, F(1, 19) = 2.67. *p* = .12). All other reliable results reported in the manuscript were obtained both when the youngest infant is and is not included. Across all infants, the average usable recording time was 4.5 hours. The total corpus was over 100 hours and consisted of 10.8 million frames. Activities and contexts were primarily recorded in the infant’s home (84.1%) but also included outdoor or group settings (10.7%), traveling in an automobile (3.6%) and other locations (1.5%). Videos from each infant were screened for privacy and accidental recordings (1.5% of total recording) and those sections were subsequently removed from the dataset. As illustrated in Fig 1b, the recordings were then downsampled to one still frame for every 5 seconds of video yielding a total of 72,150 frames that were coded for faces. Sampling at 1/5Hz is not biased to any individual or activity and appears sufficiently dense to capture major regularities: First, a coarser sampling of images at 1/10Hz yielded the same reliable patterns reported below. Second, a sampling of a different set of images at 1/5 Hz using new starting points for each of the 22 infants yielded the same proportions of images with faces for each infant (mean delta in proportion = 0.009, range delta in proportion = -0.02 to 0.09) and no reliable differences, *t*(42) = 0.32, *p* = 0.7521, across the two samplings.

For coding, the sampled frames were organized into randomly ordered sets of 100 frames. Coders were trained on a set of 9 instruction frames and were asked to answer the yes/no question “do you see a face or a part of a face in this image?”. A frame was deemed to contain a face if at least three out of four coders agreed. For 98% of the frames, at least 3 coders were in agreement that the frame did or did not contain a face. The coded measures for all frames that included faces are available at http://databrary.org/volume/99.

The sampled frames with faces were subsequently coded for the identity of the face, the distance of the face from the head-camera, and whether both eyes of the face were in view. For these measures, frames were presented to a trained team of coders in chronological order as face streams and then a second set of trained coders reanalyzed 20% of the frames. For identity, coders assigned a unique code to each individual’s face in each infant’s dataset for up to 20 different identifiable faces per infant. The cap of 20 identities was selected because beyond the five most frequently encountered identities other faces occurred very infrequently. Identities were also not determined for the faces in frames in which the face was occluded, blurry, too small or in a crowd of 4 or more faces and thus difficult to identify. Identities for faces in media (product packaging, television, books) were also not determined. All these faces were included in the total face count but not the unique identities count; the proportion of not-identified faces (for all the reasons listed above) was very small, less than .01 of all face frames (maximum for an individual participant was .022), and the proportion of not-identified faces was not significantly correlated with age, R^2^ = .07, F(1, 20) = 2.68. *p* = .12. Agreement on identity across the two sets of coders was 97.8%.

To estimate the distance of each face from the head camera, coders were asked to match faces and face parts with face size templates. Seven templates were generated by capturing a median female adult face [42] at increments of 1 foot from the head camera; coder agreement was 98.11%. Finally, coders were also asked whether both eyes were visible for all faces in the sampled frames. Agreement across coders was 93.2%.

## Results

**Fig 2:**
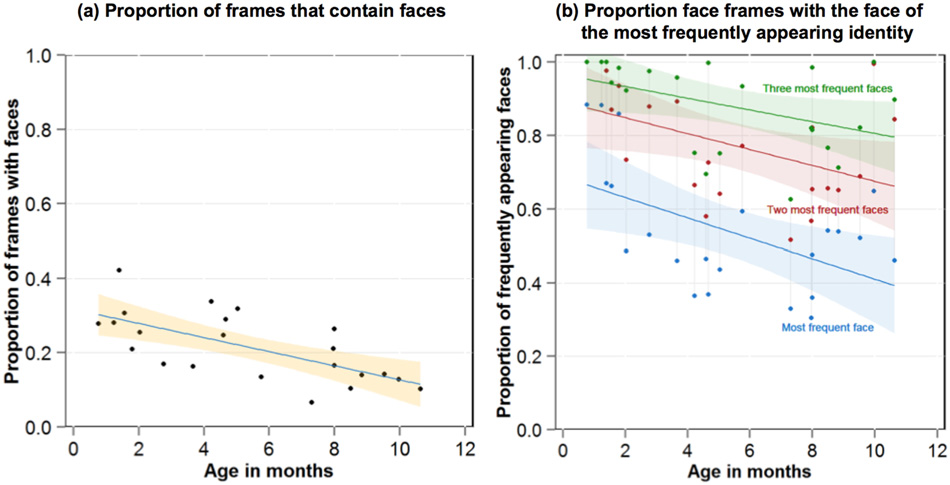
Frequencies and identities of faces in the environment. The proportion of frames that contain faces in each infant’s corpus as a function of the age of the infant and the 95% confidence interval around the best fitting line (a); The proportion of face frames containing the face of the most (blue), the two most (red) and the three most (green) frequently appearing individuals (b). Shaded regions indicate the 95% confidence interval around the best fitting line.

Fig 2a shows the proportion of faces in the sampled frames for each infant as a function of their age. The frequency of faces in the infant-perspective scenes declined markedly with age (R^2^ = .42, F(1, 20) = 16.11, *p* < .001; Spearman’s correlations were also calculated for linear fits to ensure that the conclusions remained unchanged under assumptions of non-linearity as well linearity; these analyses provide the same pattern of reliable results.). These proportions also provide an estimate of the amount of time that faces are visually available to infants: For the youngest infants, nearly 15 minutes out of every recorded hour of scenes included a face, but for the oldest infants only about 5 minutes of recorded scenes per hour included a face. Thus, the visual world of very young infants is relatively dense with face information and this density declines with age.

The mean number of unique people (identities) in the infants’ face corpora was 8.2 (SD = 5.5) and varied from as few as 2 to 20 unique faces (the maximum possible given the coding system). The total number of unique faces was not correlated with age (R^2^ = .02, F(1, 20) = 1.49, *p* = .24). However, for each infant, the faces of some individuals appeared much more frequently than others. Fig 2b shows for each infant the proportion of scenes with faces that were the faces of the three most frequently appearing individuals. The proportion of all faces that were the most frequent, (R^2^ = .23, F(1, 20) = 7.41, *p* < .05), the two most frequent (R^2^ = .16, F(1, 20) = 5.24, *p* < .05), and the three most frequent (R^2^ = .14, F(1, 20) = 4.51, *p* < .05) individuals declined reliably with age. For all infants, a few faces were encountered with great frequency and the faces of other individuals were encountered more sparingly. Within the present sample, there is a gradual expansion of the range of unique faces prior to the first birthday. The present corpora of faces may underestimate a developmental expansion as most recordings took place in the home and older infants may be expected to have more out-of-home activities that would bring them into contact with others. However, this expansion may consist primarily of extending the low-frequency tail of the distribution. The present results suggest that a few high frequency individuals dominate the available faces for very young infants and perhaps also for well into the first year of an infant’s life.

**Fig 3:**
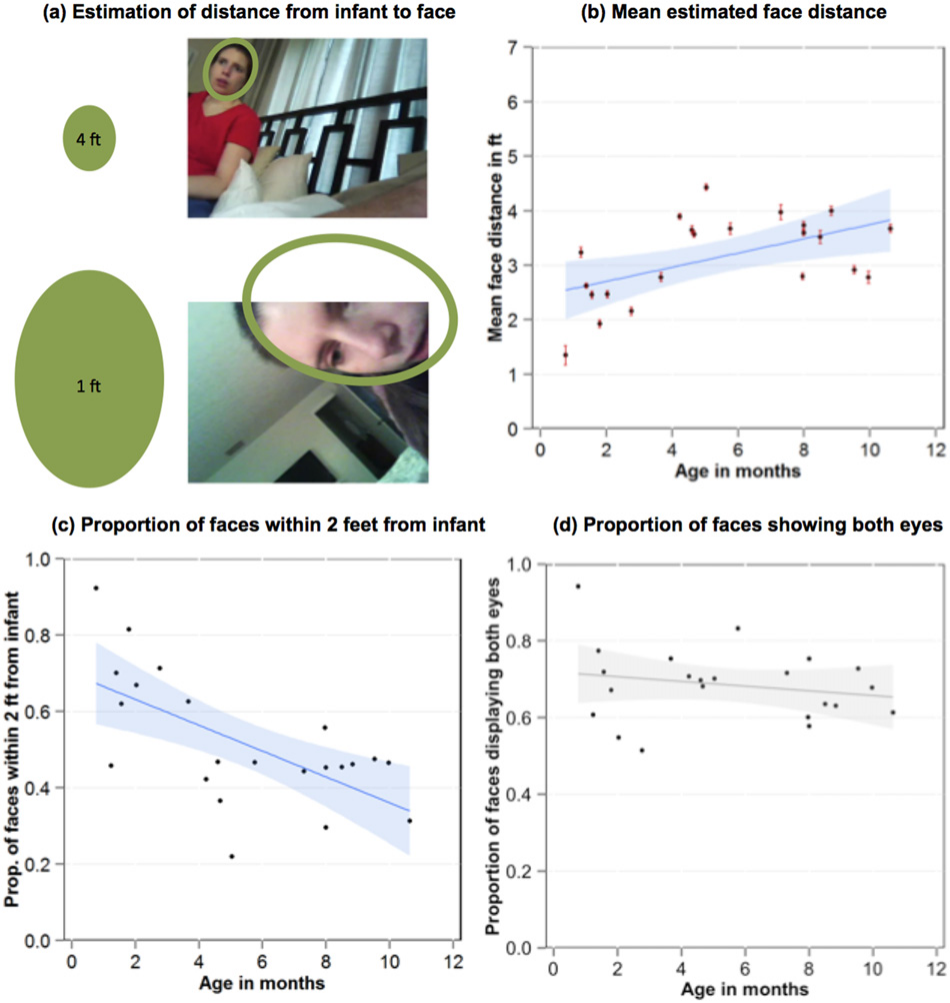
Quality of face input available to infants. Illustration of the face templates used to estimate distance of face from camera by size (a). The mean estimated distance of faces in each infant’s corpus as a function of infant age (b). The error bars represent the standard error. The proportion of faces in each infant’s corpus that was estimated to be closer than 2 feet to the infant as a function of age (c). The proportion of faces that displayed both eyes in each infant’s corpus as a function of infant age (d). The shaded areas in b, c and d show the 95% confidence interval around the best fitting line.

Fig 3b shows the mean estimated proximity of the faces to the head camera in each infant’s corpus of faces and that visually available faces were particularly close to the youngest infants. The means of the estimated distances of faces for each infant varied from under 2 feet to more than 4 feet and increased reliably with age (R^2^ = .26, F(1, 20) = 8.24, *p* < .05) with younger infants being more likely to be exposed, on average, to nearer faces and older infants to farther faces. Because the average distance of faces may obscure the relative frequency of near and far faces and because near faces may provide the most critical information, particularly for young infants, Fig 3c shows the proportion of faces in each infant’s corpus of faces that were less than two feet from the head camera. We chose the 2-foot boundary based on an estimation of the distance needed to detect the iris of the eyes in an adult face given measures of newborn acuity [26, 27]. As is evident in Fig 3b, the majority of faces fall within this boundary for the youngest infants. The proportion of faces closer than 2 feet declined reliably with age (R^2^ = .37, F(1, 20) = 13.61, *p* = .01). Thus early infancy appears especially dense with faces that present potentially optimal visual information for the visual acuity of young infants.

Fig 3d shows the proportion of faces for each infant that were frontal or near frontal views in the sense of having both eyes in view. For all infants, faces with both eyes in view comprised more than 50% of the recorded faces and this measure of the quality of face information did not vary with age (R^2^ = .04, F(1, 20) = .88, *p* = .36). Thus the faces in view present the frontal view and fit the face template hypothesized to innately attract infant visual attention [6].

**Fig 4:**
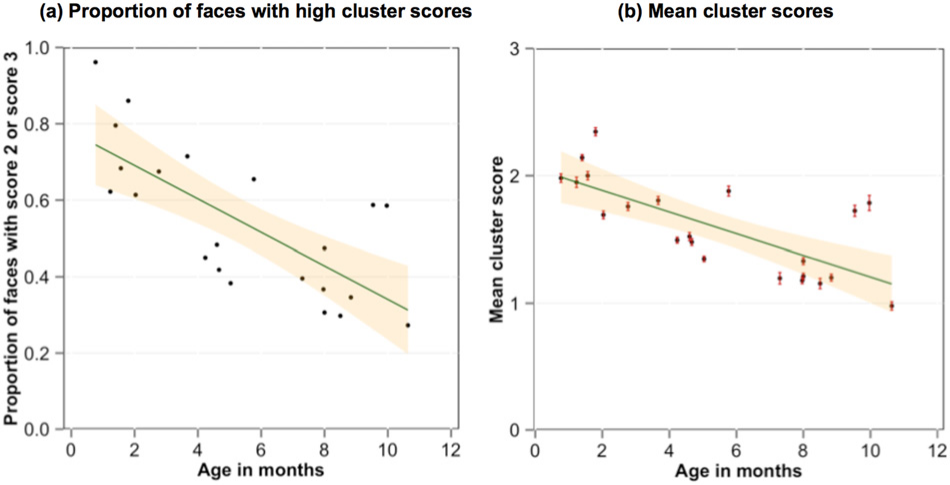
Signature properties of face experiences. The proportion of faces in each infant’s corpus of faces that showed at least 2 of the 3 signature properties of being one of the top 3 individuals, within 2 feet and showing both eyes as a function of infant age (a). The mean number of these three signature properties (cluster scores) presented by each face in each infant’s face corpus as a function of the age of the infant and the standard error around those means (b). The shaded areas show the 95% confidence interval around the best fitting line.

These analyses suggest three signature properties of faces in the first year of life: the available faces (1) belong to a few individuals, (2) appear close to the infant, and (3) display both eyes. We gave each face in the corpus of faces a cluster score that comprised one point for each of the three signature properties (thus scores could vary from 0, none of the properties, to 3, all of the properties). Fig 4a shows the proportion of recorded faces for each infant with a score of at least 2; Fig 4b shows the mean cluster score for each infant. As is apparent, the likelihood that the recorded faces contained at least 2 of the 3 cluster properties was very high for the youngest infants and declined with age (R^2^ = .54, F(1, 20) = 23.28, *p* < .001) as did the mean cluster score, (R^2^ = .52, F(1, 20) = 23.7, *p* < .001).

In summary, the analyses of the collected corpora of infant-perspective scenes yield two main conclusions about developmental changes in the quantity and quality of faces in the visual environment over the first year: First, faces are highly frequent—in a quarter or more of infant-perspective scenes—for very young infants. The quantity of faces in infant-perspective scenes declines markedly over the first year. Second, for all infants, available faces are predominantly the frontal (or near frontal) view of a very few individuals and are spatially close to the infant. Within the present corpus, variation around this prototype increases slowly as a function of age; variability in the quality of faces is thus more tightly constrained in earlier than later visual environments.

## Discussion

**Fig 5:**
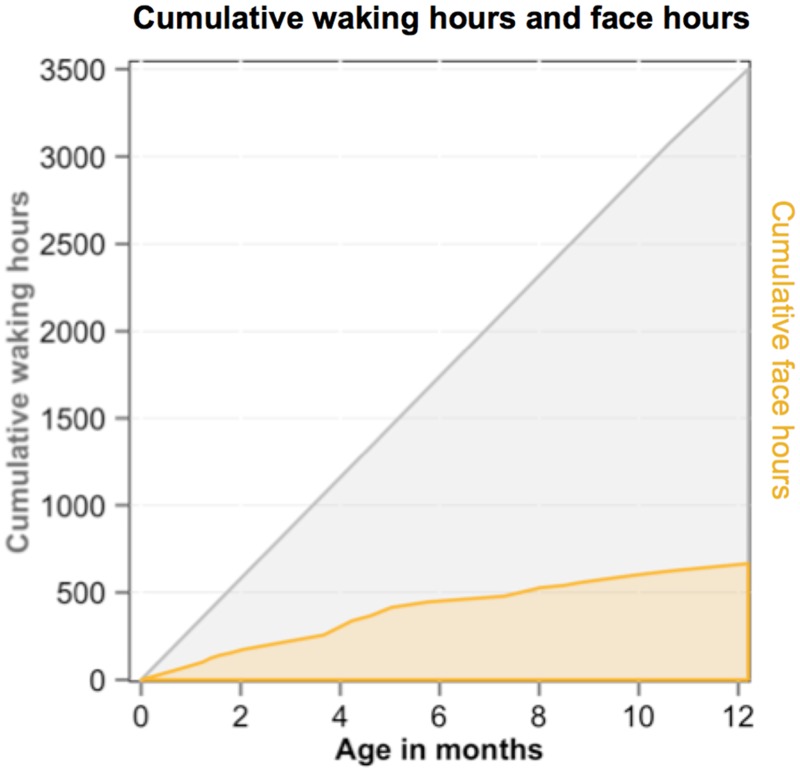
Dense early face experiences. The estimated cumulative waking hours (grey) and hours of faces (yellow). See text for estimation procedure.

The theoretical significance of the present findings is clarified by considering how the cumulative hours of face availability relate to the cumulative waking hours of infants over the first year. To estimate these cumulative effects, we treated our sample of 22 infants as a theoretically normative infant and used published norms of the number of waking hours of infants in their first year of life to estimate cumulative awake hours [17]. Fig 5 shows the estimated number of cumulative waking hours over the first year from these norms and the estimated cumulative hours of faces from the collected infant perspective scenes. The cumulative hours of expected face exposure increase at a much slower rate than the number of waking hours. This means that very young infants’ waking hours are more densely filled with faces than are older infants’ whose waking hours are apparently more often filled with other kinds of visual entities. By the time an infant is 3 months old, she has been awake for 800 hours. By the present estimate, more than a quarter of those hours, about 210, consisted of faces directly in front of her. By 11 months, an infant will have been awake for about 3100 hours and about 620 of those hours, a fifth of cumulated visual experience, will be scenes including faces. Thus the developmental trajectory is one in which face experiences are front-loaded, more frequent earlier than later in visual experience. Although we did not measure the frequency of other kinds of visual experiences, the estimated cumulative exposure in Fig 5 strongly implies that no other single object category—bottle, cup, dog, or chair—is likely to be experienced with the same frequency of faces in the early months. Thus, these early faces may have a disproportionate influence—not just on face processing—but on the early development of the visual system more generally.

These front-loaded faces are not just frequent but have prototypical characteristics that appear especially constrained in the first three months of life; the visual world of young infants is replete with repetitions of the faces of a few individuals that are visually large and show both eyes. Are these prototypical properties of early face experiences in some way vital to the development of specialized face processing? An affirmative answer is suggested by studies of face perception in individuals who were deprived of typical visual experiences in the first three months of life. Individuals who were institutionalized as infants and lacked typical exposure to the faces of a few caretakers [43] and individuals who were born with cataracts removed by 3 months of age [44], show deficits as adults in configural face processing, although other aspects of face processing may be normal. Configural processing sets face perception apart from non-face object recognition. That is, face processing, more than the visual processing of other objects, depends on a the integration of features across the whole [45] and on the spatial relations among the features [46]. The hypothesis, then, is that the distinctive properties of early visual experiences—highly frequent and repeated exposures at close proximity to the frontal views of the faces of very few individuals—are essential to the development of configural face processing.

This hypothesis is developmentally interesting on two grounds. First, configural face processing is a relatively late development; even minimal feature integration is not evident until 7 months [2, 47] and configural processing as usually defined is not achieved until late in childhood [44, 48, 49]. Thus, the role of these signature properties of early face experiences may not be in training configural face processing per se but rather in earlier visual developments that are part of the developmental chain of events that lead to these later developments (see [44, 50] for discussions of such “sleeper effects” in brain development). Second, the function of configural face processing is generally considered to be the identification and discrimination of many different individual faces [51], a task that is not encountered by very young infants. Again, the first three months of face exposures may be critical to the development of configural face processing because they maintain, tune, or foster the development of a neural architecture that can support the *later* development of configural face processing. Finally, older infants also see mostly the faces of a few individuals (albeit at greater distances). The first 3 months may be critical to the development of human face perception not simply because of the properties of the face stimuli themselves but because, as Fig 5 suggests, faces dominate over other kinds of visual inputs; whereas in later infancy, faces are more intermingled in visual experiences with other kinds of scenes. These hypotheses require more direct tests, as well as more detailed analyses of the changing information provided by face and non-face objects in infant-perspective scenes but they contribute by illustrating the importance of describing the developmental trajectory of early visual environments in a complete account of the development of human face processing.

These present findings also inform the debate concerning an experience-driven—versus genetically-driven origin to the special properties of mature face perception and recent calls that the field move beyond its current framing [52]. Given the general importance of the information that faces convey to humans, it makes sense that human face processing is developmentally constrained. Critical species outcomes often have developmental pathways that are strongly buffered by multiple and redundant constraints so as to not be easily derailed (see [18, 53]). The present results are consistent with a role for constrained early visual environments among other potential constraints [53]. Because human infants are altricial, care by a few invested adults that brings their faces frequently close to the infant may be an evolutionarily-expected property of experience and one which is central to early developments in the visual system. Although the present sample included children from just one cultural context, and although parenting practices can vary widely, the needed care for very young infants may nearly universally constrain the range and character of expected face experiences [54, 55] making faces the predominant single class of objects for the first few months of life.

However, this conclusion does not rule out the possibility of other constraints [52], including genetically determined neural processes. One line of research that supports a genetic basis concerns the newborn bias to look at very simple “face—like” arrays consisting of two dark blobs (eyes) within a face-shaped contour [8, 9]. This neonatal bias has been interpreted in terms of an “experience-expectant innate template” [6] that directs infant attention to faces and ensures the engagement of prepared neural processes with face stimuli [56]. The present results suggest that the early faces that infants encounter fit this template in that faces are predominantly frontal views and sufficiently close and visually large that the eyes on these faces should be detectable even given young infants’ limited acuity. Up close frontal views may thereby provide an optimal start for developing specialized face processing, engaging subcortical processes and tuning cortical processes [9, 57]. The environmental constraint suggested by the present results and an innate face template fit together like a glove on a hand. Because of their immaturity, the young infant’s visual world is dense with the faces of a few up-close caregivers; these faces match the face template that in turn ensures that infants attend to those faces. In this way, a detailed study of early visual environments complements research on early intrinsic biases.
